# Epigenetic silencing of *PHYHD1* in hepatocellular carcinoma revealed by integrated multi-omics analysis

**DOI:** 10.3389/fgene.2026.1848528

**Published:** 2026-06-22

**Authors:** Tianfu Guo, Yafei Li, Tiansheng He, Hui Luo, Yuwen Liu, Binhui Xie, Zhiping Liu

**Affiliations:** 1 Key Laboratory of Innate Immunity and Chronic Inflammatory Diseases, Jiangxi Provincial Department of Education, Gannan Medical University, Ganzhou, China; 2 Key Laboratory of Prevention and Treatment of Cardiovascular and Cerebrovascular Diseases, Ministry of Education, Gannan Medical University, Ganzhou, China; 3 School of Basic Medicine, Gannan Medical University, Ganzhou, China; 4 The First Affiliated Hospital of Gannan Medical University, Ganzhou, Jiangxi, China

**Keywords:** DNA methylation, epigenetic silencing, hepatocellular carcinoma, multi-omics, Phyhd1

## Abstract

**Introduction:**

Hepatocellular carcinoma (HCC) is characterized by extensive epigenetic alterations, particularly DNA methylation. Through integrated multi-omics analysis, we identified methylation-driven genes in HCC, including PHYHD1—a previously understudied candidate whose role in hepatocarcinogenesis remains unexplored. This study aimed to validate and functionally characterize PHYHD1 in HCC.

**Methods:**

Five pairs of HCC and adjacent non-tumor tissues were analyzed using whole-genome bisulfite sequencing (WGBS), RNA-seq, and TMT-based proteomics. We identified 97,523 differentially methylated regions (DMRs), 875 differentially expressed genes (DEGs), and 225 differentially expressed proteins (DEPs). *PHYHD1* hypermethylation was confirmed by public methylation datasets, and its mRNA/protein expression was validated by RT-qPCR, Western blot, and immunohistochemistry in cell lines, patient tissues (n = 76 pairs), and a DEN/CCl_4_-induced murine HCC model. Functional effects were assessed via *in vitro* assays (CCK-8, colony formation, flow cytometry) and an *in vivo Phyhd1* knockout model.

**Results:**

Integration of methylome, transcriptome, and proteome data revealed 19 consistently altered genes enriched in metabolic pathways. *PHYHD1* was hypermethylated and downregulated at both mRNA and protein levels in HCC. However, gain- or loss-of-function of PHYHD1 had no detectable effect on proliferation, apoptosis, or tumorigenicity *in vitro* or *in vivo* (all *P* > 0.05).

**Discussion:**

Integrated multi-omics analysis identified *PHYHD1* as an epigenetically silenced gene in HCC, but functional studies indicate that its silencing does not act as a canonical driver of malignant phenotypes. Instead, *PHYHD1* hypermethylation may represent a passenger event or a context-dependent modulator that only exerts effects under specific stress conditions (e.g., hypoxia or metabolic inflammation). These findings suggest that not all methylation-driven genes in HCC are functional drivers, and they highlight the need for further investigation into passenger epigenetic alterations in tumor biology.

## 1 Introduction

Liver cancer represents a major global health challenge, ranking as the sixth most common cancer and the third leading cause of cancer-related mortality worldwide. According to estimates from the International Agency for Research on Cancer (IARC), approximately 865,000 new cases of liver cancer are diagnosed annually, resulting in nearly 757,948 deaths (Bray et al., 2024). As the predominant form of primary liver cancer, Hepatocellular carcinoma (HCC) is characterized by its high malignancy and rapid progression. The disease often remains asymptomatic in its early stages, and the lack of effective treatment options means that most patients are diagnosed at an advanced stage, contributing to a poor prognosis (Llovet et al., 2021). Therefore, a deeper understanding of the molecular mechanisms driving HCC development is critical for identifying novel biomarkers to improve early diagnosis and enable precision therapies.

Epigenetic alterations, particularly DNA methylation, serve as key contributors to tumorigenesis. As a fundamental epigenetic mechanism, DNA methylation regulates gene expression by modifying promoter activity and is involved in critical biological processes such as cell differentiation, genomic stability, and cancer development (Fu et al., 2023; Nagaraju et al., 2022). HCC exhibits a distinct methylation landscape, combining genome-wide hypo-methylation, which can promote genomic instability and proto-oncogene activation with localized promoter hyper-methylation that often silences tumor suppressor genes (Hernandez-Meza et al., 2021). These changes play a pivotal role in HCC initiation, progression, and metastasis.

Previous studies have largely relied on methylation microarray technologies (e.g., 27K or 450K arrays) to profile DNA methylation in HCC, providing important insights into its epigenetic regulation (Revill et al., 2013; Zhang et al., 2019). However, these arrays are limited to pre-defined CpG sites, leaving large portions of the methylome unexplored and potentially missing rare methylation variants and novel driver events. Moreover, most studies have focused exclusively on methylation-transcriptome relationships (Zhao et al., 2024), which are essential for bridging the gap between genetic alterations and functional protein outcomes.

Recent advances in high-throughput sequencing have made it possible to characterize methylation patterns at single-base resolution using whole-genome bisulfite sequencing (WGBS), offering an unbiased view of the HCC methylome (Lister et al., 2009). In parallel, multi-omics integration strategies now enable a systems-level exploration of HCC, combining genomic, epigenomic, transcriptomic, and proteomic data to uncover novel drivers and pathways (Zhang et al., 2014; Qiang et al., 2019). This approach has already demonstrated promise in identifying new therapeutic targets and refining HCC classification (Hernandez-Meza et al., 2021).

Despite these advances, several critical knowledge gaps remain. First, the majority of methylation studies in HCC have not integrated proteomic data, limiting their ability to link epigenetic changes to functional protein outcomes. Second, while numerous methylation-driven genes have been identified, many remain functionally uncharacterized. Third, the field lacks a systematic understanding of why some epigenetically silenced genes do not exhibit canonical tumor-suppressive functions. The present study aims to address these gaps by: (1) performing an integrated multi-omics analysis (WGBS, RNA-seq, and proteomics) of paired HCC and adjacent non-tumor tissues; (2) systematically identifying and validating methylation-driven genes; and (3) functionally characterizing a previously understudied candidate, PHYHD1, to determine its role in HCC pathogenesis.

## 2 Materials and methods

### 2.1 Clinical samples and data

This study was approved by the Ethics Committee of the First Affiliated Hospital of Gannan Medical University (Approval No. 2024495), and written informed consent was obtained from all participating patients. Between February and April 2019, we collected five pairs of fresh-frozen tissue samples, comprising HCC tissue and matched adjacent non-tumor tissue, from male patients (average age: 66 years; range: 52–78 years) with pathologically confirmed HCC. All tissue samples were immediately snap-frozen in liquid nitrogen following surgical resection and stored for subsequent multi-omics sequencing analysis. To further validate our findings, a commercially available tissue microarray (Product No.: HLivH160CS02, Shanghai Outdo Biotech Co., Ltd.) containing 76 pairs of HCC and adjacent non-tumor tissues was utilized for immunohistochemistry (IHC) validation.

Publicly DNA methylation data (Illumina HumanMethylation450K BeadChip) and RNA-seq data were downloaded from The Cancer Genome Atlas (TCGA) database (TCGA-LIHC, n = 371 HCC, n = 50 normal). Gene methylation and expression levels were further validated using the Gene Expression Omnibus (GEO) datasets (GSE136319, GSE136583, GSE112790, and GSE121248). Proteomics data were obtained from the Clinical Proteomic Tumor Analysis Consortium (CPTAC) database.

### 2.2 Animals and cell lines

Male wild-type (WT) and Phyhd1 knockout (*Phyhd1* ^*−/−*^) C57BL/6J mice (2 weeks old) were purchased from GemPharmatech Co., Ltd. (Jiangsu, China). All mice were housed under specific pathogen-free (SPF) conditions in a barrier facility at the Animal Experiment Center of Gannan Medical University, with a 12-h light/dark cycle, temperature of 24 °C ± 2 °C, and relative humidity of 50% ± 5%. All animal experiments were approved by the Institutional Animal Care and Use Committee of Gannan Medical University (Approval No. 2024473).

The human normal hepatic stellate cell line LX-2 and HCC cell lines (SMMC-7721, MHCC97-H, Huh-7, and BEL-7402) were obtained from the Cell Bank of the Chinese Academy of Sciences (Shanghai, China). LX-2 and MHCC97-H cells were cultured in high-glucose DMEM; SMMC-7721, BEL-7402, and Huh-7 cells were maintained in RPMI 1640 medium. All media were supplemented with 10% FBS and 1% penicillin-streptomycin. Cells were cultured at 37 °C in a humidified incubator with 5% CO_2_. All cell lines were regularly tested for *mycoplasma* contamination, and their identities were authenticated by short tandem repeat (STR) profiling.

### 2.3 Experimental methods

#### 2.3.1 Mouse HCC model establishment

Hepatocarcinogenesis was induced in 15-day-old male WT and *Phyhd1* ^−/−^ mice by a single intraperitoneal (i.p.) injection of diethylnitrosamine (DEN) at a dose of 25 mg/kg body weight. After 1 week, mice received weekly i.p. injections of 10% carbon tetrachloride (CCl_4_) in olive oil at 0.5 mL/kg for a total of 22 weeks. Mice were euthanized by cervical dislocation at the experimental endpoint. Livers were excised, and visible surface tumor nodules (diameter ≥1 mm) were counted. The maximum tumor diameter was measured, and the liver-to-body weight ratio (liver weight/body weight × 100%) was calculated. Liver tissues were snap-frozen or fixed in 4% paraformaldehyde for histological analysis.

#### 2.3.2 Multi-omics sequencing and data analysis

**FIGURE 1:**
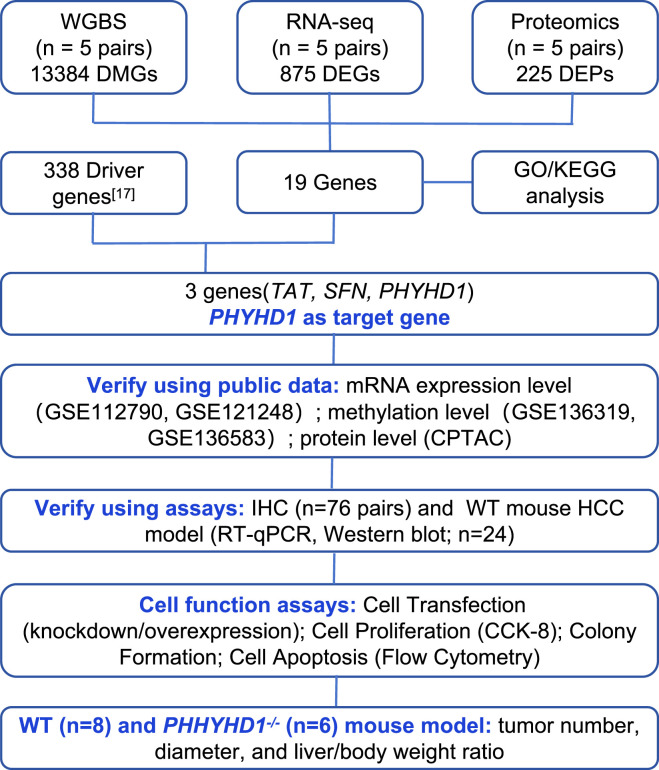
Workflow of the study. From five paired human HCC samples, WGBS, RNA-seq, and proteomics identified 13,384 DMGs, 875 DEGs, and 225 DEPs, respectively. Their intersection yielded 19 genes, which were subjected to GO/KEGG enrichment analysis. Intersecting these 19 genes with 338 known driver genes produced three genes (TAT, SFN, PHYHD1), and PHYHD1 was selected as the target. Public datasets (GSE112790, GSE121248, GSE136319, GSE136583, CPTAC) were used for verification. Experimental validation included IHC (76 pairs), WT mouse HCC model (RT-qPCR, Western blot; n = 24), *in vitro* functional assays (proliferation, colony formation, apoptosis), and *in vivo* comparison of WT (n = 8) and *Phyhd1* ^*−/−*^ (n = 6) mice for tumor number, diameter, and liver/body weight ratio.

Five pairs of HCC and adjacent non-tumor tissues underwent WGBS, RNA-seq, and quantitative proteomics analysis. The overall workflow of this study is illustrated in Figure 1.

##### 2.3.2.1 WGBS

Genomic DNA was bisulfite-converted using the EZ DNA Methylation-Gold™ Kit (Zymo Research, United States). Libraries were sequenced on an Illumina HiSeq X Ten platform (PE150). Raw data quality-checked with FastQC (v0.11.9) and trimmed with Trimmomatic (v0.39). Clean reads were aligned to the human reference genome (hg19) using Bismark (v0.22.3) to extract cytosine methylation calls (Krueger and Andrews, 2011). Differentially methylated regions (DMRs) were identified using the DSS package (v2.42.0) in R with thresholds set at |Δβ| ≥ 0.25 and false discovery rate (FDR) < 0.05 (Wu et al., 2015).

##### 2.3.2.2 RNA-seq

Total RNA was isolated using TRIzol® reagent (Invitrogen, United States). Sequencing libraries were constructed with the NEBNext® Ultra™ RNA Library Prep Kit (NEB, United States) and sequenced on an Illumina NovaSeq 6,000 platform (PE150). Raw reads were quality-controlled and filtered using Fastp (v0.23.2). High-quality clean reads were aligned to the hg19 genome using HISAT2 (v2.2.1). Transcript assembly and abundance estimation were performed with StringTie (v2.1.5). Differentially expressed genes (DEGs) were identified using the edgeR package (v4.0.16) applying a generalized linear model (GLM), with significance thresholds set at |log_2_ (fold change)| ≥ 1.5 and FDR < 0.05.

##### 2.3.2.3 Proteomics

Proteins were extracted from tissues, quantified using a BCA assay (Thermo Scientific, 23,225), and digested with trypsin following standard protocols (reduction with DTT, alkylation with IAA). Resulting peptides were labeled using TMT reagents (Thermo Scientific). Labeled samples were pooled and fractionated by high-pH reverse-phase chromatography using a RIGOL L3000 HPLC system with a Waters BEH C18 column. LC-MS/MS analysis was performed on an EASY-nLC 1200 UHPLC system coupled to a Q Exactive HF-X mass spectrometer in data-dependent acquisition (DDA) mode. Raw data were searched against the UniProt human protein database using Proteome Discoverer software (v2.2, Thermo Scientific) with specified search parameters. Protein identification was filtered at 1% FDR at the peptide-spectrum match (PSM) and protein levels. Differential protein expression analysis was performed using the limma package in R, with DEPs defined by |log_2_ (fold change)| ≥ 1 and FDR < 0.05.

#### 2.3.3 Bioinformatic analysis

Functional enrichment analysis, including Gene Ontology (GO) and Kyoto Encyclopedia of Genes and Genomes (KEGG) pathway analysis, was performed for the identified DMGs, DEGs, and DEPs using the DAVID bioinformatics resource, with an FDR < 0.05 considered significant. Protein-protein interaction (PPI) networks were constructed using the STRING database (minimum interaction score > 0.7) and visualized using Cytoscape software (v3.6.1) (Franceschini et al., 2013). Hub genes within the networks were identified using the cytoHubba plugin, selecting the top 10 nodes by connectivity (Chin et al., 2014).

#### 2.3.4 Molecular biology experiments

##### 2.3.4.1 RT-qPCR

Total RNA was extracted using the TransZol Up Plus RNA Kit (TransGen, China). cDNA was synthesized from DNase I-treated RNA using the PrimeScript RT reagent Kit (TaKaRa, Japan). Quantitative PCR was performed using SYBR Premix Ex Taq II (TaKaRa, Japan) on a QuantStudio 7 Flex system (Applied Biosystems, United States). Relative mRNA expression levels were calculated using the 2^(-ΔΔCT)^ method with β-actin as the endogenous control. Primer specificity was confirmed by BLAST analysis. All reactions were performed in triplicate.

##### 2.3.4.2 Western blotting

Proteins were extracted using RIPA lysis buffer containing PMSF, quantified by BCA assay, separated by SDS-PAGE, and transferred to PVDF membranes. After blocking, membranes were incubated overnight at 4 °C with primary antibodies against PHYHD1 (abcam, ab181232; 1:1,000) and β-Actin (Proteintech, 66009-1-Ig; 1:5,000), followed by incubation with HRP-conjugated secondary antibodies. Signals were detected using ECL substrate and analyzed with ImageJ software, normalized to β-Actin.

##### 2.3.4.3 Immunohistochemistry (IHC)

Paraffin-embedded sections underwent antigen retrieval, peroxidase blocking, and blocking with normal serum before incubation with anti-PHYHD1 antibody (1:200) overnight at 4 °C. Staining was developed using DAB after incubation with an HRP-polymer secondary antibody, followed by hematoxylin counterstaining. Staining intensity and the percentage of positive cells were scored independently by two blinded pathologists (Jin et al., 2018).

#### 2.3.5 Cell function assays

##### 2.3.5.1 Cell transfection assay

To modulate PHYHD1 expression, transfection was performed using Lipofectamine™ 3,000 reagent (Invitrogen, United States) according to the manufacturer’s protocol. The pRP [CMV]-3xFLAG hPHYHD1 overe-xpression plasmid was transfected into MHCC97-H cell lines (low endogenous PHYHD1 expression), while SMMC-7721 cells were transfected with specific short hairpin RNA (shRNA) targeting PHYHD1 (high endogenous expression). Control groups included cells transfected with a blank vector or a scrambled negative shRNA. Cells were harvested 48 h post-transfection, and transfection efficiency was confirmed by Western blot analysis (>70% efficiency was achieved).

##### 2.3.5.2 Cell proliferation assay (CCK-8)

Cell proliferation was assessed using the CCK-8 assay. Cells from each group were seeded into 96-well plates at a density of 3 × 10^3^ cells per well (n = 5 replicates per group). At 0, 24, 48, and 72 h post-transfection, 10 µL of CCK-8 reagent (Dojindo, Japan) was added to each well, followed by incubation at 37 °C for 2 h. The absorbance at 450 nm was measured using a microplate reader (BioTek, United States), and cell growth curves were generated based on the optical density (OD) values. Each experiment was independently repeated three times.

##### 2.3.5.3 Colony formation assay

Stably transfected cells were plated in 6-well plates at a low density of 500 cells per well and cultured under standard conditions (37 °C, 5% CO_2_) for 14 days, with the medium refreshed every 3 days. After the incubation period, cells were gently washed with PBS, fixed with 4% paraformaldehyde for 15 min, and stained with 0.1% crystal violet for 20 min. The plates were then rinsed under running water and air-dried at room temperature. Colonies containing more than 50 cells were counted after scanning the images. The experiment was independently repeated three times.

##### 2.3.5.4 Cell apoptosis analysis by flow cytometry

Apoptosis was evaluated 48 h after transfection using an Annexin V-APC/7-AAD Apoptosis Detection Kit (KeyGEN BioTECH, China) according to the manufacturer’s instructions. Briefly, harvested cells were stained and analyzed on a BD FACS Celesta flow cytometer (BD, United States). Data processing was performed using FlowJo software (v10.6.2). The total apoptosis rate was defined as the sum of early apoptotic (Annexin V-APC^+^/7-AAD^-^) and late apoptotic (Annexin V-APC^+^/7-AAD^+^) cell populations.

### 2.4 Statistical analysis

All experiments were independently repeated at least three times. Data are presented as mean ± standard error of the mean (SEM). Normality was assessed by Shapiro-Wilk test, and equality of variances by Levene’s or Bartlett’s test. Comparisons between two groups were performed using Student’s t-test (paired or unpaired, as appropriate), with Welch’s correction when variances were unequal. For multi-group comparisons, one-way analysis of variance (ANOVA) followed by Tukey’s post hoc test was used; when variances were unequal, Welch’s ANOVA with Games-Howell post hoc test was applied. For longitudinal data with repeated measurements (e.g., CCK-8 assays), two-way repeated measures ANOVA followed by Sidak’s multiple comparisons test was used to assess differences between groups across time points. Survival curves were generated using the Kaplan-Meier method, and differences were assessed by the log-rank test. Correlations between normally distributed variables were analyzed using Pearson’s correlation coefficient; otherwise, Spearman’s rank correlation was used. A two-tailed P-value < 0.05 was considered statistically significant. All analyses were performed using GraphPad Prism (version 9.0) and R software (version 4.3.0).

## 3 Results

### 3.1 Multi-omics profiling reveals extensive epigenetic and expression alterations in HCC

**FIGURE 2:**
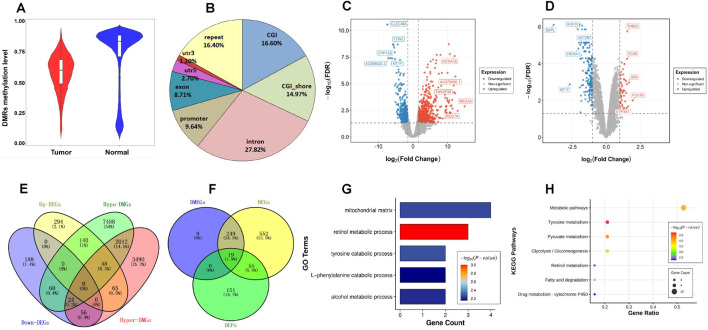
Multi-omics integration identifies core methylation-driven genes in HCC. **(A)** DMR methylation levels in HCC versus adjacent non-tumor tissues. **(B)** Proportions of DMRs in different areas. **(C,D)** Volcano plots DEGs **(C)** and DEPs **(D)** in HCC. Red and blue dots represent significantly up- and downregulated molecules, respectively. **(E)** Venn diagram illustrating the overlap between Hypo-DMGs, hyper-DMGs, up-DEGs and down-DEGs. **(F)** Integration of methylome, transcriptome, and proteome data identifies 19 consistently altered genes across all three omics layers. **(G)** Gene Ontology (GO) enrichment analysis. **(H)** KEGG pathways enrichment analysis.

We performed an integrated multi-omics analysis on five paired HCC and adjacent non-tumor tissues using whole-genome bisulfite sequencing (WGBS), RNA sequencing (RNA-seq), and TMT-based quantitative proteomics. WGBS data revealed a global hypomethylation trend in HCC tissues (Figure 2A). We identified 97,523 differentially methylated regions (DMRs), including 51,234 hypermethylated and 46,289 hypomethylated DMRs in HCC compared to adjacent tissues (FDR < 0.05, |Δβ| ≥ 0.25). These DMRs were predominantly enriched in intronic regions (27.82%), CpG islands (16.60%), and CpG island shores (14.97%) (Figure 2B; Supplementary Table S1), corresponding to 13,384 differentially methylated genes (DMGs). Transcriptome analysis identified 875 differentially expressed genes (DEGs; 547 upregulated, 328 downregulated) (Figure 2C; Supplementary Table S2). Proteomic profiling revealed 225 differentially expressed proteins (DEPs; 53 upregulated, 172 downregulated) (Figure 2D; Supplementary Table S3). These results demonstrate widespread dysregulation at the epigenetic, transcriptomic, and proteomic levels in HCC.

### 3.2 Identification of core methylation-regulated genes through multi-omics integration

Integration of methylome and transcriptome data identified 268 differentially methylated and expressed genes (DMEGs), comprising 80 hypermethylated and downregulated genes and 188 hypomethylated and upregulated genes (Figure 2E). Functional enrichment analysis indicated their involvement in extracellular matrix organization and metabolic processes (Supplementary Figures S1A, S1B). Protein-protein interaction (PPI) network analysis highlighted key hub genes among these DMEGs (Supplementary Figures S1C, S1D). Further integration of transcriptome and proteome data identified 74 genes consistently differentially expressed at both mRNA and protein levels (Figure 2F), which were significantly enriched in metabolic pathways (Supplementary Figures S1E-S1H). Cross-analysis of all three omics layers pinpointed 19 genes with concordant alterations in methylation, mRNA, and protein levels (13 downregulated, 6 upregulated) (Figure 2F; Supplementary Table S4). These multi-omics core genes showed significant enrichment in metabolic pathways, including retinol and tyrosine metabolism (Figures 2G,H; FDR < 0.05 for both).

### 3.3 Candidate gene selection

**FIGURE 3:**
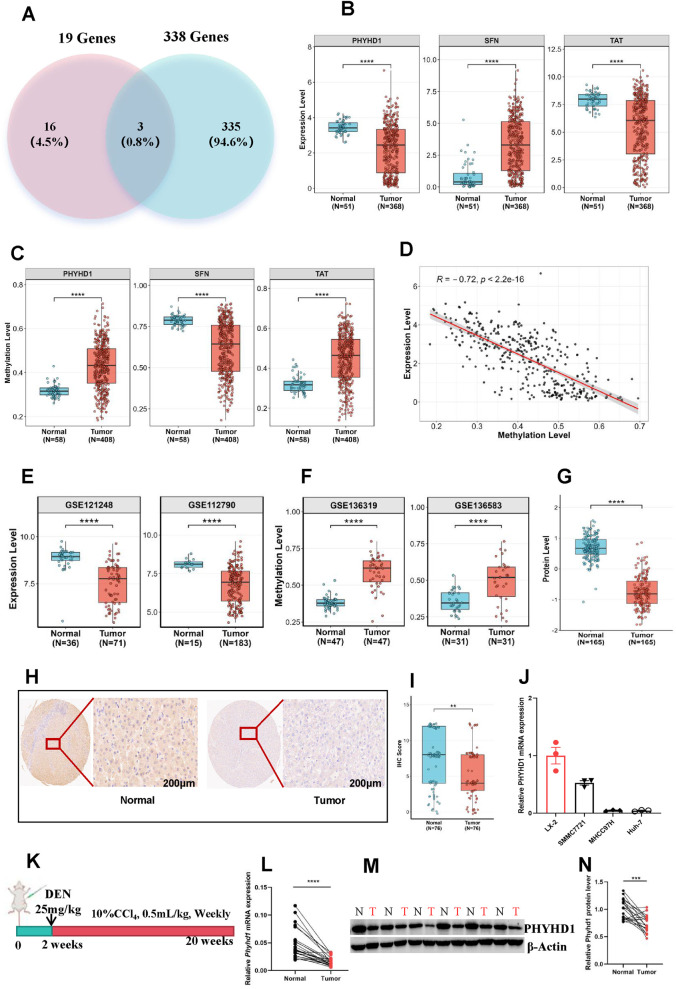
Screening of candidate genes. **(A)** Overlap between the 19 multi-omics core genes and 338 methylation driven genes from TCGA. **(B,C)** mRNA expression **(B)** and methylation levels **(C)** of TAT, SFN, and *PHYHD1* in HCC versus normal tissues from TCGA. **(D)** Correlation between *PHYHD1* methylation and its mRNA expression. **(E,F)** Validation of lower mRNA **(E)** and higher promoter methylation **(F)** in GEO datasets. **(G)** CPTAC protein levels. **(H,I)** IHC representative images and scores. **(J)** *PHYHD1* expression in cell lines. **(K–N)** DEN/CCl_4_ mouse model showing reduced Phyhd1 mRNA and protein (paired t-test, n = 24 pairs). *P < 0.05; **P < 0.01; ***P < 0.001; ****P < 0.0001.

We intersected the 19 multi-omics core genes with 338 methylation-driven genes identified from TCGA analysis (Zhu et al., 2021), which prioritized *TAT, SFN,* and *PHYHD1* for further investigation (Figure 3A). Analysis of TCGA data confirmed a hypermethylated/low-expression pattern for *TAT* and *PHYHD1*, and a hypomethylated/high-expression pattern for SFN in HCC (Figures 3B,C). While *TAT* and *SFN* have established roles in HCC (Fu et al., 2010; Li et al., 2024). the function of *PHYHD1* remains unclear. A significant negative correlation was observed between *PHYHD1* methylation and its expression (Pearson’s *R* = −0.72, *P* < 0.001) (Figure 3D), leading us to focus subsequent studies on PHYHD1.

### 3.4 *PHYHD1* is hypermethylated and downregulated in HCC

Validation using independent datasets confirmed higher methylation levels (GSE136319, GSE136583) and lower mRNA expression (GSE112790, GSE121248) of *PHYHD1* in HCC tissues compared to non-tumor tissues (Figures 3E,F; all *P* < 0.001). Consistent with this, CPTAC data showed significantly lower *PHYHD1* protein levels in HCC (Figure 3G). Immunohistochemistry (IHC) revealed that *PHYHD1* protein was localized in both the cytoplasm and nucleus, and its expression was significantly reduced in HCC tissues (Figures 3H,I; *P* < 0.001, n = 76 pairs). At the cellular level, RT-qPCR showed higher *PHYHD1* expression in LX-2 hepatic stellate cells than in several HCC cell lines (SMMC-7721, MHCC97-H, Huh-7) (Figure 3J; *P* < 0.01 for all comparisons). In a DEN/CCl_4_-induced mouse HCC model, Phyhd1 mRNA and protein levels were significantly lower in tumor tissues compared to adjacent non-tumor liver tissues (Figures 3K–N; *P* < 0.001). These consistent findings across models and platforms establish *PHYHD1* as a hyper-methylated and downregulated gene in HCC.

### 3.5 Modulation of *PHYHD1* expression does not affect HCC cell proliferation or apoptosis

**FIGURE 4:**
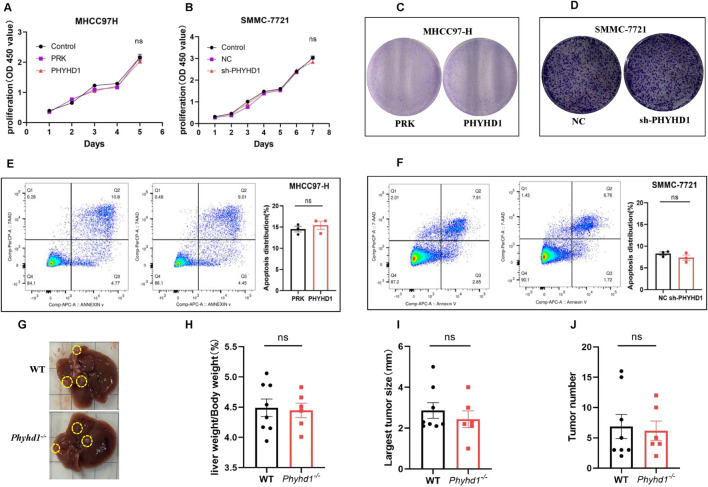
Functional assessment of PHYHD1. **(A,B)** CCK-8 proliferation after overexpression **(A)** and knockdown **(B)** (Two-way repeated measures ANOVA). **(C,D)** Colony formation. **(E,F)** Apoptosis by flow cytometry. **(G)** Representative livers from WT and *Phyhd1* ^*−/−*^ mice. **(H–J)** Quantification of liver/body weight ratio, tumor number, and largest tumor size (Mann-Whitney U test).

Transfection efficiency was confirmed by Western blot (>70% knockdown/overexpression achieved). CCK-8 assays showed that altering *PHYHD1* expression failed to significantly affect cell proliferation (Figures 4A,B; all *P* > 0.05). Similarly, colony formation assays revealed no detectable changes in clonogenic ability upon *PHYHD1* over-expression or knockdown (Figures 4C,D; *P* > 0.05 for both). Flow cytometry analysis of apoptosis indicated that the rates of early and late apoptosis showed no appreciable alteration (Figures 4E,F; *P* > 0.05 for both). These *in vitro* results suggest that *PHYHD1* had no detectable direct regulation of HCC cell proliferation or apoptosis under the tested conditions.

### 3.6 Phyhd1 knockout does not influence HCC development in mice

To assess the role of *PHYHD1 in vivo*, we subjected *Phyhd1* ^*−/−*^ mice (n = 6) and wild-type (WT, n = 8) controls to a DEN/CCl_4_-induced HCC carcinogenesis model. Compared to WT mice, *Phyhd1* ^*−/−*^ mice showed no significant differences in tumor number (WT: 6.88 ± 5.64 vs. KO: 6.17 ± 3.92, *P* = 0.79), maximum tumor diameter (WT: 2.86 ± 1.09 mm vs. KO: 2.43 ± 1.00 mm, *P* = 0.46), or liver-to-body weight ratio (WT: 4.49% ± 0.41% vs. KO: 4.45% ± 0.30%, *P* = 0.83) (Figures 4G–J; Supplementary Figure S2). These *in vivo* findings are consistent with the *in vitro* results, indicating that under the present experimental conditions, loss of *Phyhd1* did not markedly impact HCC development.

## 4 Discussion

This study represents an integrated multi-omics analysis combining WGBS, RNA-seq, and proteomics to identify methylation-driven genes in HCC, with a particular focus on the previously uncharacterized gene *PHYHD1*. We successfully pinpointed 19 candidate genes consistently dysregulated across the epigenetic, transcriptional, and translational levels, and confirmed that *PHYHD1* is hypermethylated and downregulated in HCC across multiple independent datasets, human tissues, and a mouse model. To our knowledge, this is one of the few studies to systematically validate the epigenetic silencing of *PHYHD1* in HCC. Surprisingly, in our experimental systems (cell lines and DEN/CCl_4_ model), altering *PHYHD1* expression did not significantly affect HCC cell proliferation, apoptosis, or *in vivo* tumorigenicity. This seemingly contradictory finding prompts a reevaluation of the complex role of epigenetic silencing events in HCC initiation and progression.

The integrated multi-omics strategy employed here effectively overcomes the limited coverage of traditional methylation microarrays. WGBS provides a genome-wide methylation profile at single-base resolution, and integrating this with transcriptome and proteome data significantly improves the accuracy of identifying driver events. Consistent with previous reports (Yan et al., 2021; Zhao et al., 2024), we observed a genome-wide hypo-methylation pattern in HCC tissues. Among our 19 candidate genes, several are well-established HCC-related genes. For instance, *SFN* promotes the malignant progression of HCC cells by enhancing AKT phosphatase activity through disrupting the PHLPP2-AKT interaction (Li et al., 2024), while *TAT* induces mitochondria-dependent apoptosis in HCC cells by promoting cytochrome C release, thereby activating caspase-9 and PARP (Fu et al., 2010). The identification of these known genes strongly validates the reliability of our screening approach, justifying our focus on the previously understudied gene, *PHYHD1*.

*PHYHD1* encodes a structural analog of phytanoyl-CoA hydroxylase (PHYH/PAHX). This enzyme acts on fatty acids, localizes to peroxisomes, and is mutated in certain neurological disorders (Wanders et al., 2001). Genomic variations in *PHYHD1* are associated with altered levels of nucleotide metabolites, including guanine, 5-methylcytosine, and 5-methyluracil (Bar et al., 2020). In various cell lines, endogenous and recombinant *PHYHD1* are localized in both the nucleus and cytoplasm. In contrast, its closest homolog, the 2-oxoglutarate-dependent dioxygenase (2OGDD) PHYH, is peroxisomal (Jansen et al., 1996). Cells lacking *PHYHD1* also exhibit reduced efficiency in utilizing glucose and maltose. Pathway analysis links *PHYHD1* to cell division and RNA metabolism proteins (particularly those involved in mRNA splicing), and suggests potential roles in mRNA transport and transcription (Ala-Nisula et al., 2023). Current research on *PHYHD1*’s role in disease is limited: it has been associated with T cell differentiation and effector T cell function in mice (Furusawa et al., 2016); altered methylation and transcription of *PHYHD1* have been observed in non-functioning pituitary adenomas (Cheng et al., 2019),and a genome-wide association study identified *PHYHD1* as a novel genetic determinant of blood metabolites in chronic kidney disease (Rhee et al., 2022). However, its role and mechanism in cancer, particularly in HCC initiation and progression, had not been reported prior to our study.

In our validation, we consistently observed *PHYHD1* hyper-methylation and downregulation across multiple independent datasets, human tissues, and mouse models. Subsequent functional experiments, however, revealed that neither over-expression nor knockdown of *PHYHD1* affected HCC cell proliferation, apoptosis, or *in vivo* tumorigenicity. This “epigenetic silencing versus functional loss paradox” can be explained in several ways. First, *PHYHD1* hypermethylation may be a “passenger event”—a consequence of global epigenetic dysregulation in HCC—rather than a direct driver of tumorigenesis. Recent large-scale analyses have suggested that many epigenetic alterations in cancer arise as bystander effects rather than functional drivers (Dimitrakopoulos et al., 2021). Second, *PHYHD1* function may be highly context-dependent, with its role becoming critical only under specific conditions, such as metabolic stress or immune microenvironment interactions, which were not replicated in our standard experimental settings. Third, functional redundancy or compensatory mechanisms might offset the direct phenotypic impact of *PHYHD1* loss. Fourth, as a putative oxygen sensor associated with carbohydrate metabolism (Ala-Nisula et al., 2023), *PHYHD1* may primarily function under hypoxic conditions typical of the tumor microenvironment. Notably, developmental signaling pathways such as Hedgehog are also intricately linked to oxygen sensing and hypoxic responses in the tumor microenvironment, where their aberrant activation promotes cancer stem cell maintenance and therapeutic resistance (Kumar et al., 2025). Whether *PHYHD1* intersects with such pathways under hypoxic or inflammatory stress remains an open question for future investigation.

Despite the lack of a direct functional effect in our proliferation assays, the consistent epigenetic silencing of *PHYHD1* in HCC suggests potential clinical utility as a biomarker. Specifically, methylated *PHYHD1* DNA could potentially be detected in circulating tumor DNA (ctDNA) from HCC patient serum samples, serving as a non-invasive early diagnostic marker for HCC. Future studies should evaluate whether PHYHD1 methylation in liquid biopsies correlates with tumor burden, treatment response, or patient prognosis. Furthermore, the role of PHYHD1 in the context of chronic liver inflammation and metabolic dysfunction warrants investigation. Chronic hepatitis B (CHB) frequently coexists with metabolic dysfunction-associated steatotic liver disease (MASLD) in HCC patients, creating a complex metabolic and inflammatory microenvironment (Li and Luo, 2025). The interplay between *PHYHD1* silencing and these metabolic disturbances remains unexplored. Future studies should examine whether *PHYHD1* expression correlates with markers of oxidative stress, lipid metabolism, or inflammatory signaling in HCC tissues.

This study has several limitations. First, the initial WGBS cohort was small (n = 5 pairs); although we validated our findings using multiple public datasets (TCGA; CPTAC; GEO), a larger prospective cohort would strengthen our conclusions. Second, the DEN/CCl_4_-induced mouse HCC model does not fully recapitulate the complex heterogeneity, metabolic dysfunction, and immune microenvironment of human HCC, particularly the context of chronic viral hepatitis or metabolic liver disease. Third, our functional assays were limited to proliferation and apoptosis under standard culture conditions; we did not assess potential effects on cell migration, invasion, metabolic reprogramming, or response to oxidative stress. Fourth, the specific molecular and biochemical functions of *PHYHD1*—including its putative role as an oxygen sensor and its involvement in carbohydrate metabolism—remain incompletely characterized. Fifth, we did not perform metabolic profiling of PHYHD1-manipulated cells, which might reveal alterations in glucose utilization or lipid metabolism not captured by proliferation assays (Ala-Nisula et al., 2023).

Recent studies have highlighted the importance of multi-omics approaches in understanding HCC pathogenesis. For example, integrated analyses have revealed that many methylation-driven genes in HCC function in metabolic pathways rather than canonical cell cycle regulation (Wali et al., 2025). Our findings align with this emerging paradigm, as the 19 core genes we identified were significantly enriched in retinol and tyrosine metabolism pathways. Moreover, the observation that an epigenetically silenced gene (*PHYHD1*) lacks canonical tumor-suppressive functions raises the possibility that some methylation events in HCC may represent bystander or passenger alterations rather than true drivers.

## 5 Conclusion

In conclusion, this study identified a panel of 19 potential methylation-driven genes in HCC through integrated multi-omics analysis and, for the first time, systematically validated *PHYHD1*’s epigenetic silencing and explored its function. Our results suggest that the frequent epigenetic silencing of *PHYHD1* in HCC does not directly drive the malignant phenotype under standard culture conditions. Instead, *PHYHD1* may act as a context-dependent gene that exerts effects under specific stress conditions (e.g., hypoxia, metabolic stress) or indirectly contributes to HCC progression by regulating the tumor microenvironment. This finding not only deepens our understanding of the complexity of epigenetic regulation in HCC but also highlights future research directions. Future studies should explore *PHYHD1*’s specific roles in hepatic metabolic homeostasis, oxidative stress resistance, and tumor microenvironment regulation using models that better mimic physiological and pathological conditions, such as systems involving metabolic disturbance, hypoxia, or cell co-culture. Additionally, the potential utility of *PHYHD1* methylation as a non-invasive biomarker for early HCC detection warrants investigation in prospective cohorts.

## Data Availability

The original contributions presented in the study are publicly available. The multi-omics data (including WGBS, RNA_seq, and TMT proteomics) generated in this study can be found in the China National Center for Bioinformation / Beijing Institute of Genomics, Chinese Academy of Sciences (CNCB) with the BioProject accession number PRJCA064938, at https://ngdc.cncb.ac.cn/bioproject/browse/PRJCA064938.
