# Supplementary material for: Epigenetic silencing of PHYHD1 in hepatocellular carcinoma revealed by integrated multi-omics analysis
Source: Front Genet. 2026 Jun 22;17:1848528. doi: 10.3389/fgene.2026.1848528 (PMC13333458; doi:10.3389/fgene.2026.1848528)

- **Supplementary Figure 1. Enrichment and network analysis of methylation-regulated genes.**

- **(A, B)** GO (A) and KEGG (B) enrichment analyses of 268 differentially methylated and expressed genes (DMEGs).
- **(C, D)** PPI network of 268 DMEGs (C) and the top 10 hub genes identified by connectivity (D).
- **(E, F)** GO (E) and KEGG (F) enrichment analyses of 74 genes consistently altered at both mRNA and protein levels.
- **(G, H)** PPI network of the 74 multi-omics consistent genes (G) and the corresponding top 10 hub genes (H).

A

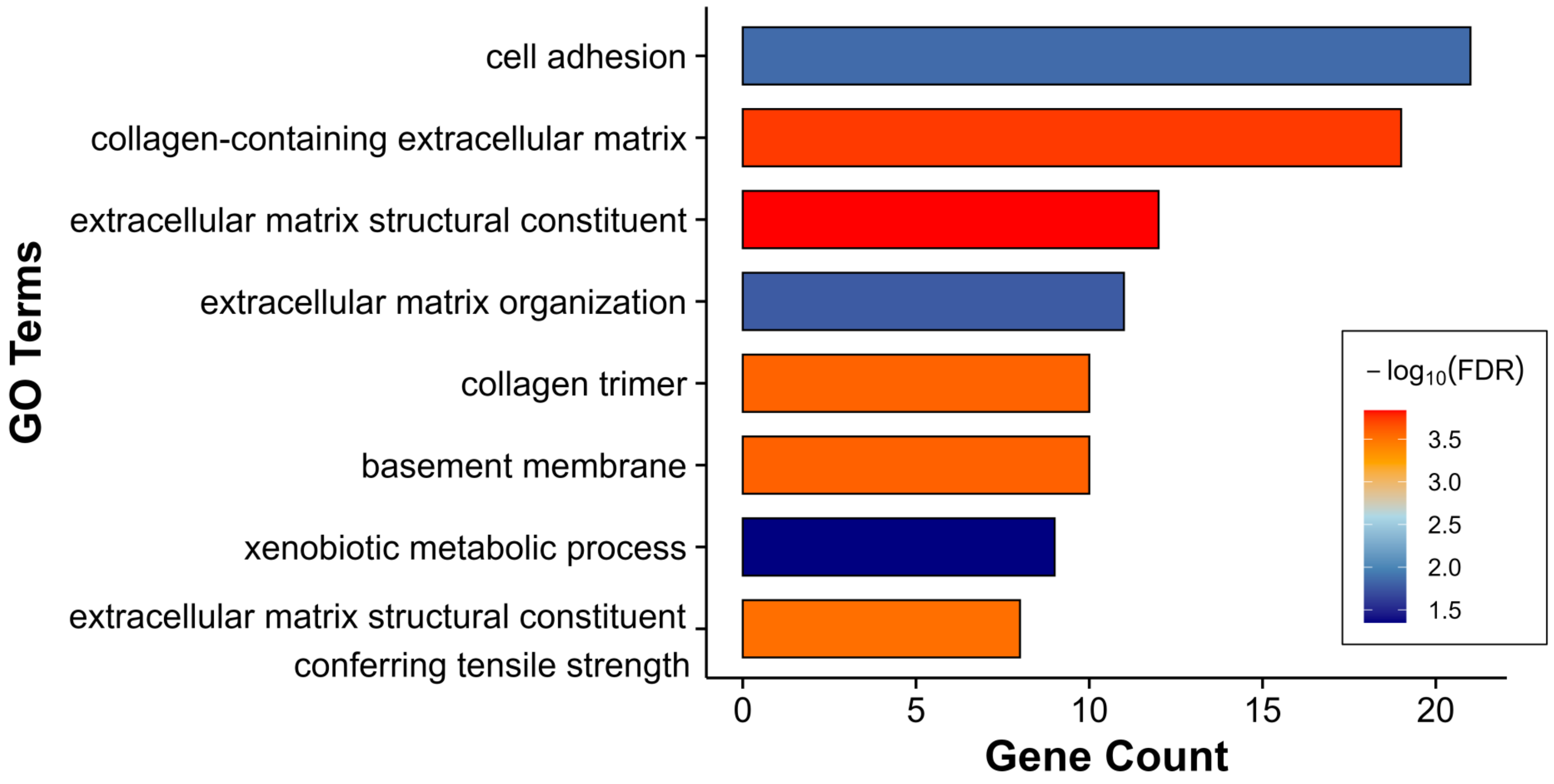

GO Terms
cell adhesion
collagen-containing extracellular matrix
extracellular matrix structural constituent
extracellular matrix organization
collagen trimer
basement membrane
xenobiotic metabolic process
extracellular matrix structural constituent conferring tensile strength
0
5
10
15
20
Gene Count
− log10(FDR)
3.5
3.0
2.5
2.0
1.5

B

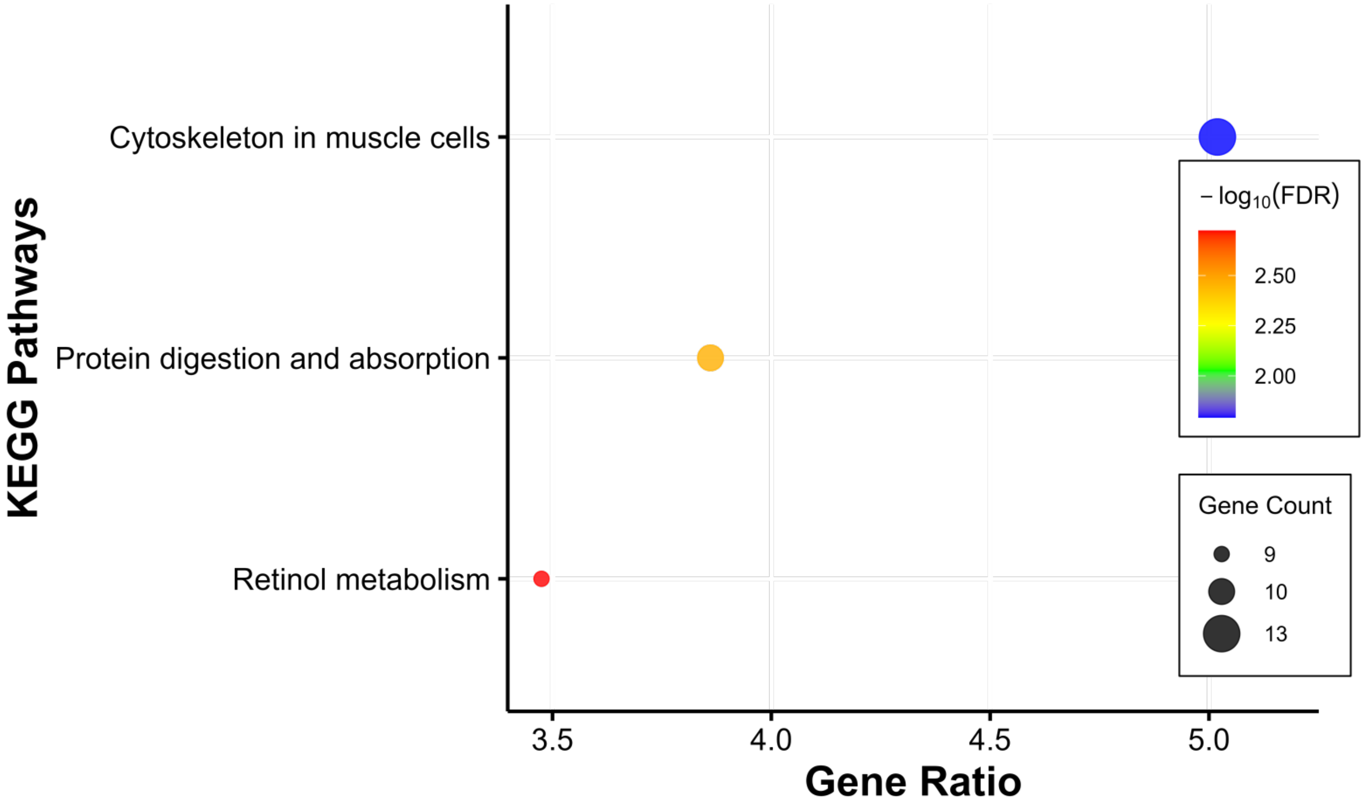

KEGG Pathways
Cytoskeleton in muscle cells
Protein digestion and absorption
Retinol metabolism
3.5
4.0
4.5
5.0
Gene Ratio
− log10(FDR)
2.50
2.25
2.00
Gene Count
9
10
13

C

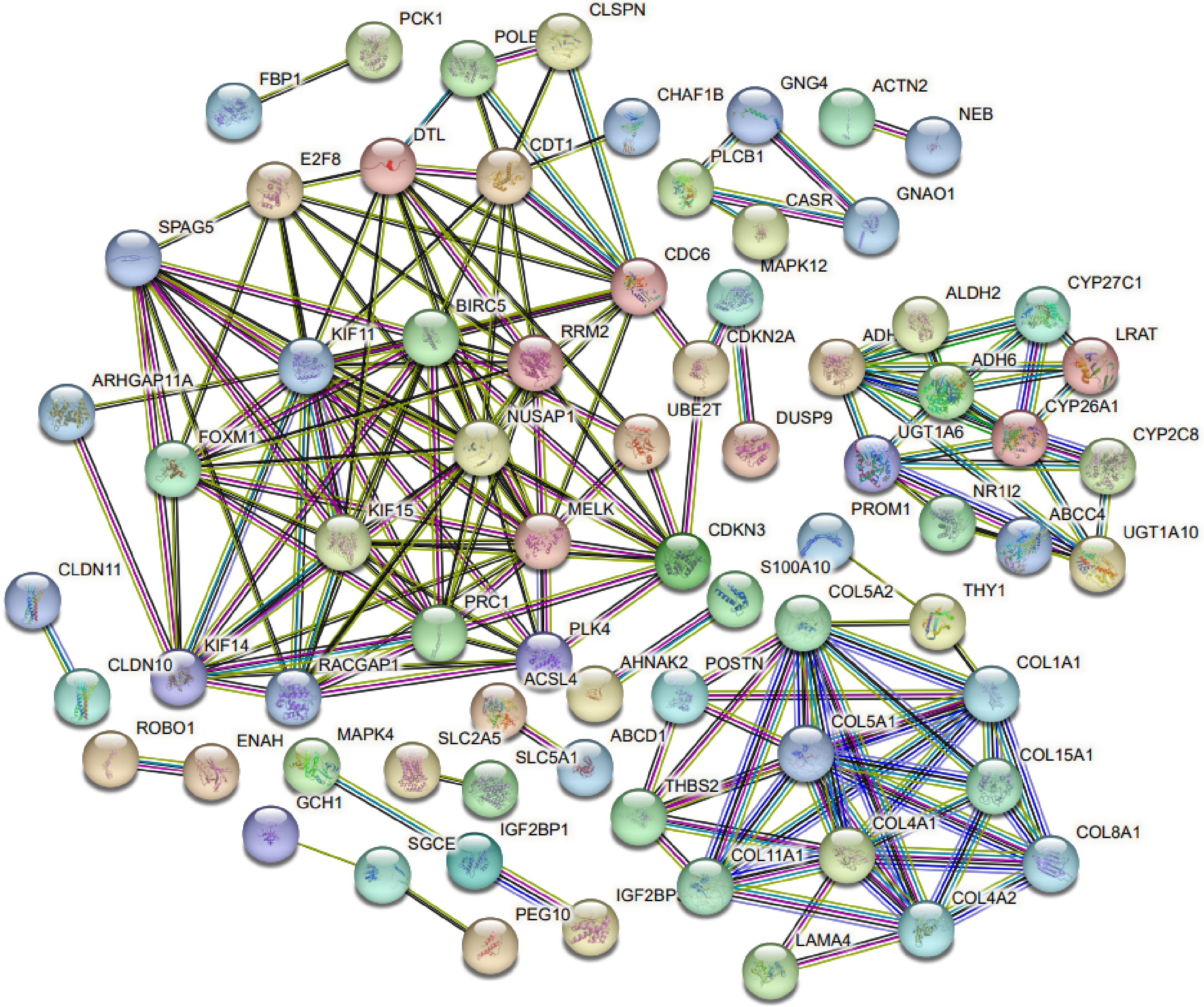

PCK1
CLSPN
POLE
FBP1
CHAF1B
GNG4
ACTN2
NEB
DTL
E2F8
CDT1
PLCB1
CASR
GNAO1
SPAG5
MAPK12
CDC6
BIRC5
KIF11
RRM2
CDKN2A
ALDH2
CYP27C1
LRAT
ADH6
ARHGAP11A
UBE2T
CYP26A1
NUSAP1
DUSP9
UGT1A6
CYP2C8
FOXM1
NR1I2
PROM1
ABCC4
KIF15
MELK
CDKN3
UGT1A10
CLDN11
S100A10
COL5A2
THY1
PRC1
PLK4
KIF14
CLDN10
RACGAP1
AHNAK2
POSTN
ACSL4
COL1A1
COL5A1
ROBO1
ENAH
MAPK4
SLC2A5
ABCD1
SLC5A1
COL15A1
THBS2
GCH1
IGF2BP1
COL4A1
SGCE
COL8A1
COL11A1
IGF2BP
COL4A2
PEG10
LAMA4

D

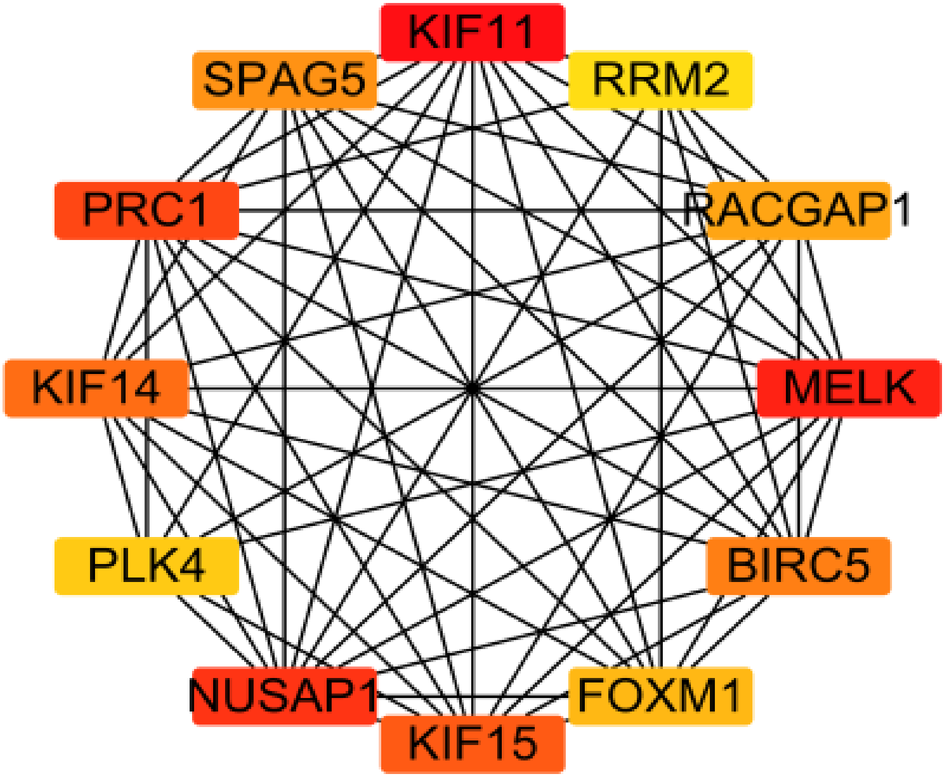
KIF11
SPAG5
RRM2
PRC1
RACGAP1
KIF14
MELK
PLK4
BIRC5
NUSAP1
FOXM1
KIF15

E

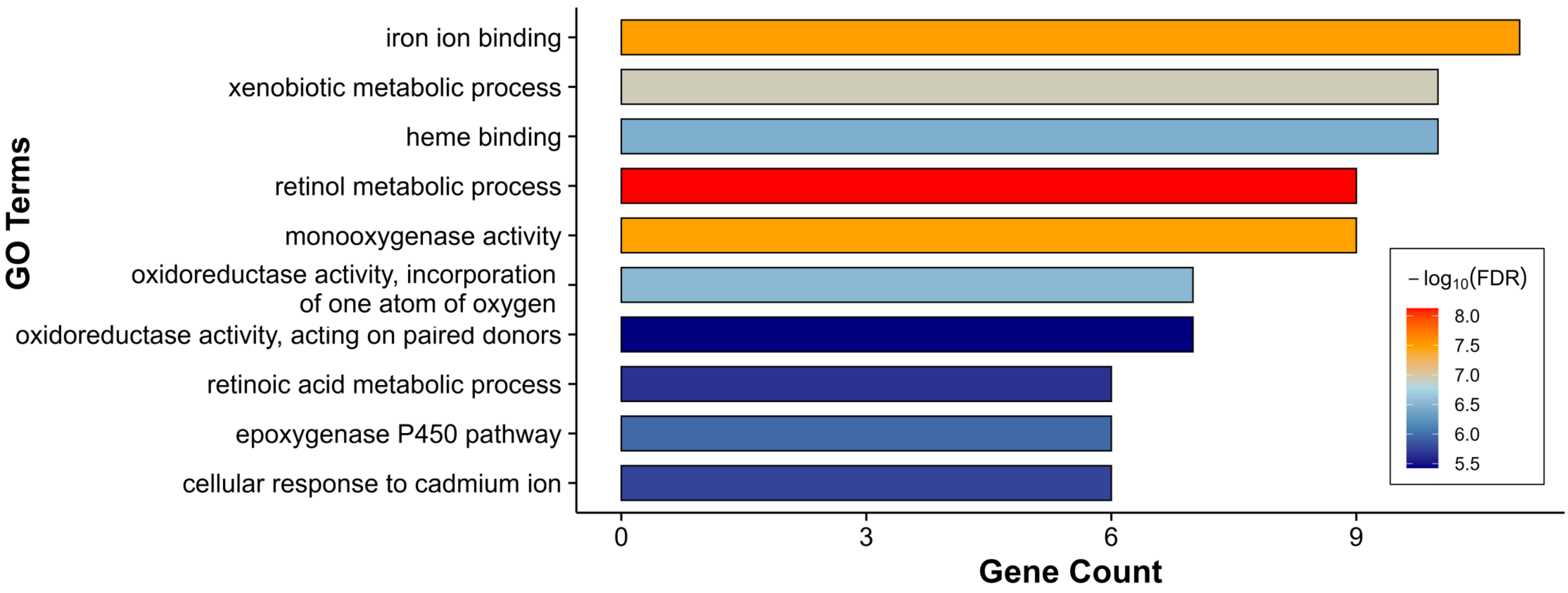

GO Terms
iron ion binding
xenobiotic metabolic process
heme binding
retinol metabolic process
monooxygenase activity
oxidoreductase activity, incorporation of one atom of oxygen
oxidoreductase activity, acting on paired donors
retinoic acid metabolic process
epoxygenase P450 pathway
cellular response to cadmium ion
0
3
6
9
Gene Count
−log10(FDR)
8.0
7.5
7.0
6.5
6.0
5.5

F

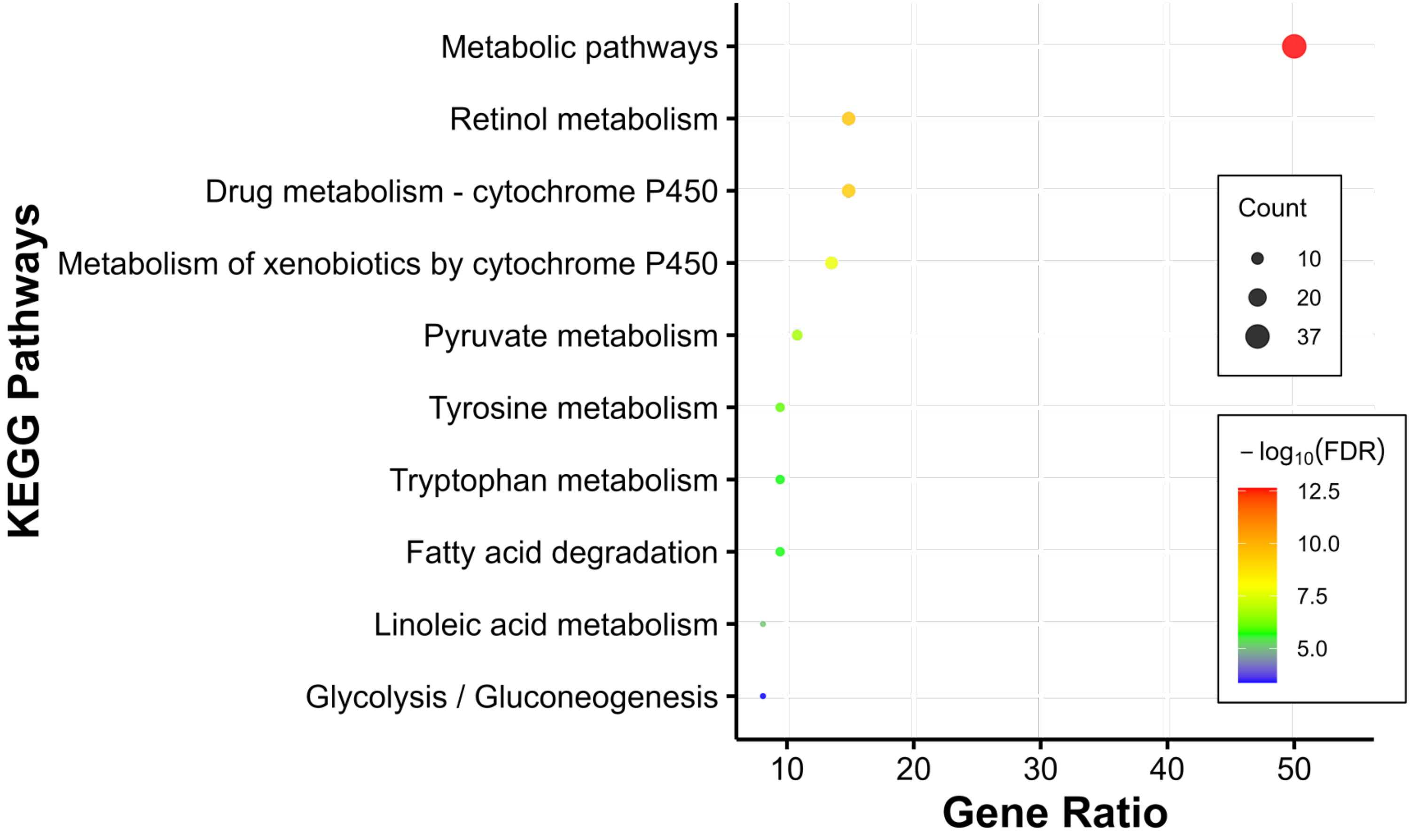

KEGG Pathways
Metabolic pathways
Retinol metabolism
Drug metabolism - cytochrome P450
Metabolism of xenobiotics by cytochrome P450
Pyruvate metabolism
Tyrosine metabolism
Tryptophan metabolism
Fatty acid degradation
Linoleic acid metabolism
Glycolysis / Gluconeogenesis
10
20
30
40
50
Gene Ratio
Count
10
20
37
− log10(FDR)
12.5
10.0
7.5
5.0

G

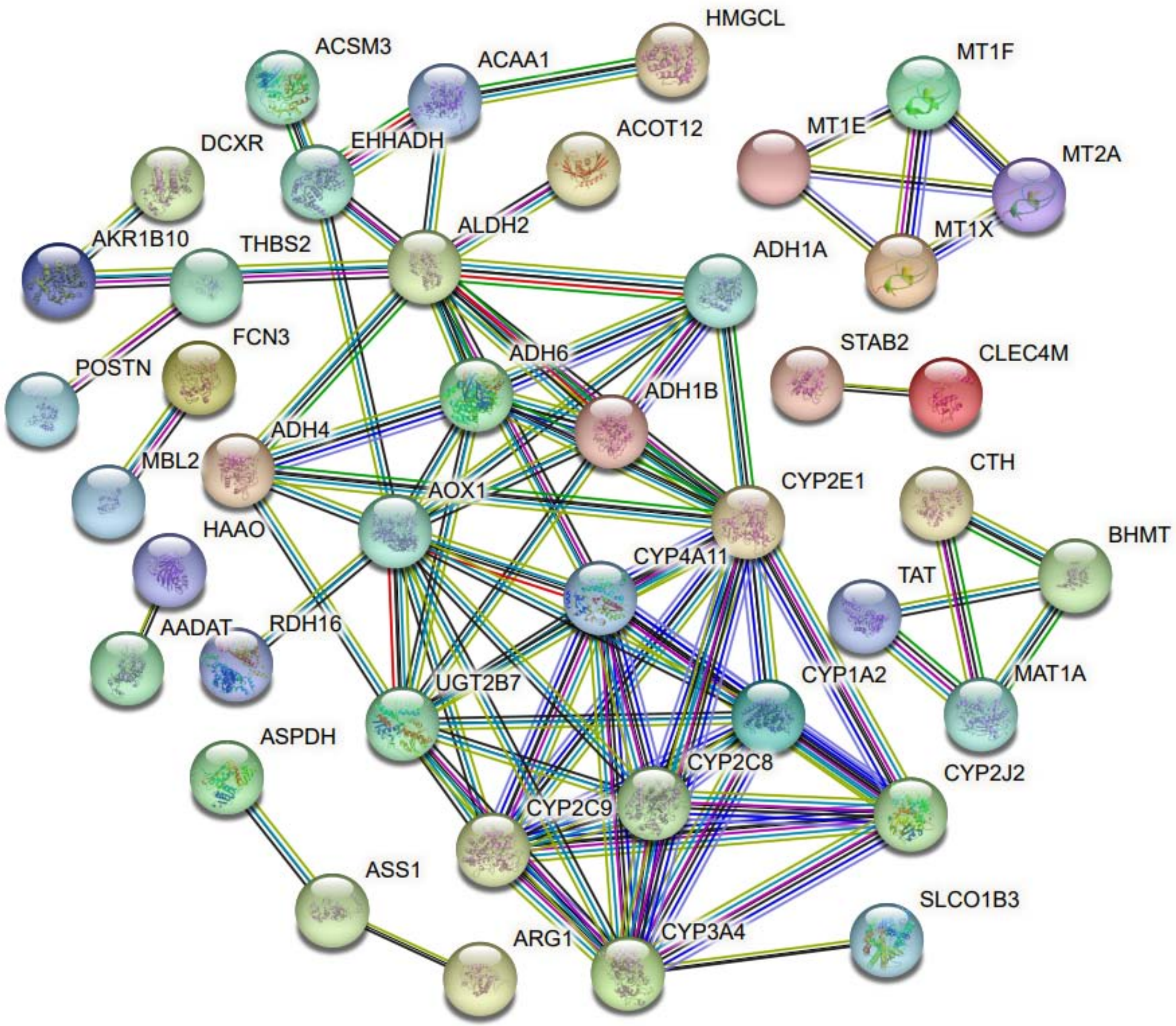
HMGCL
ACSM3
ACAA1
MT1F
ACOT12
MT1E
DCXR
EHHADH
MT2A
ALDH2
MT1X
AKR1B10
THBS2
ADH1A
STAB2
CLEC4M
FCN3
ADH6
POSTN
ADH1B
ADH4
MBL2
CTH
AOX1
CYP2E1
HAAO
BHMT
CYP4A11
TAT
AADAT
RDH16
CYP1A2
MAT1A
UGT2B7
ASPDH
CYP2C8
CYP2J2
CYP2C9
ASS1
SLCO1B3
ARG1
CYP3A4

H

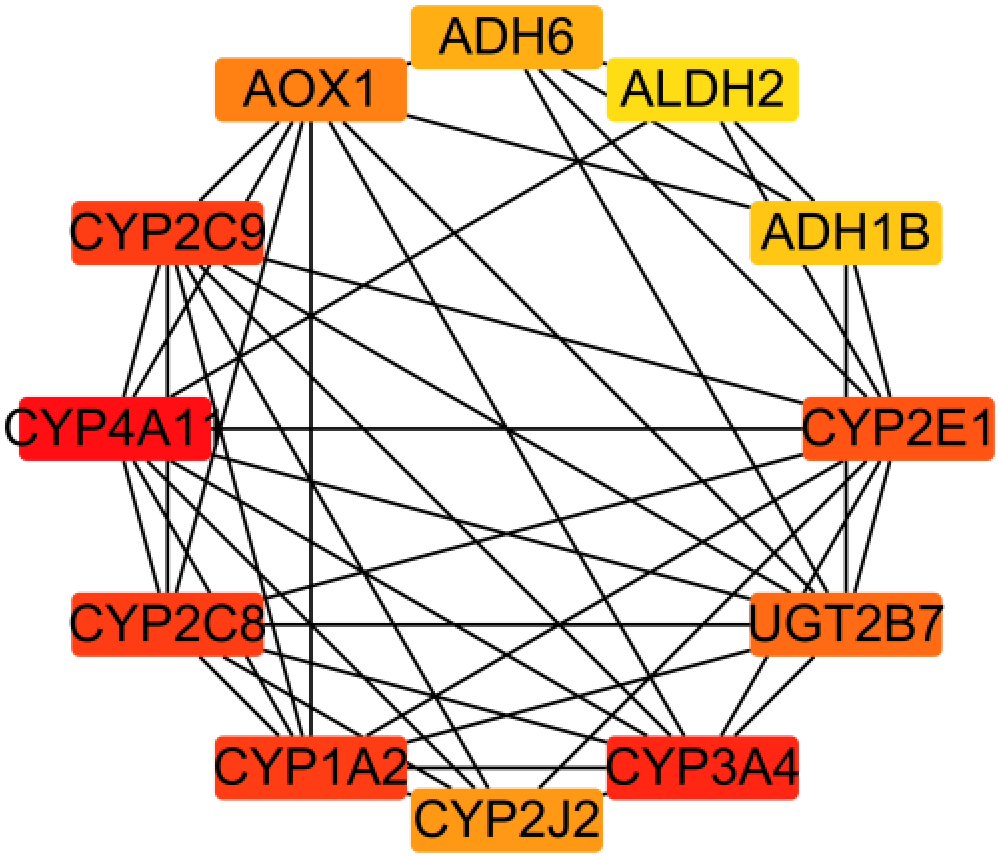

ADH6
AOX1
ALDH2
CYP2C9
ADH1B
CYP4A11
CYP2E1
CYP2C8
UGT2B7
CYP1A2
CYP3A4
CYP2J2

- **Supplementary Figure 2. Representative livers from wild-type (WT) and *Phyhd1*$^{-/-}$ mice after DEN/CCl_4-induced hepatocarcinogenesis.**

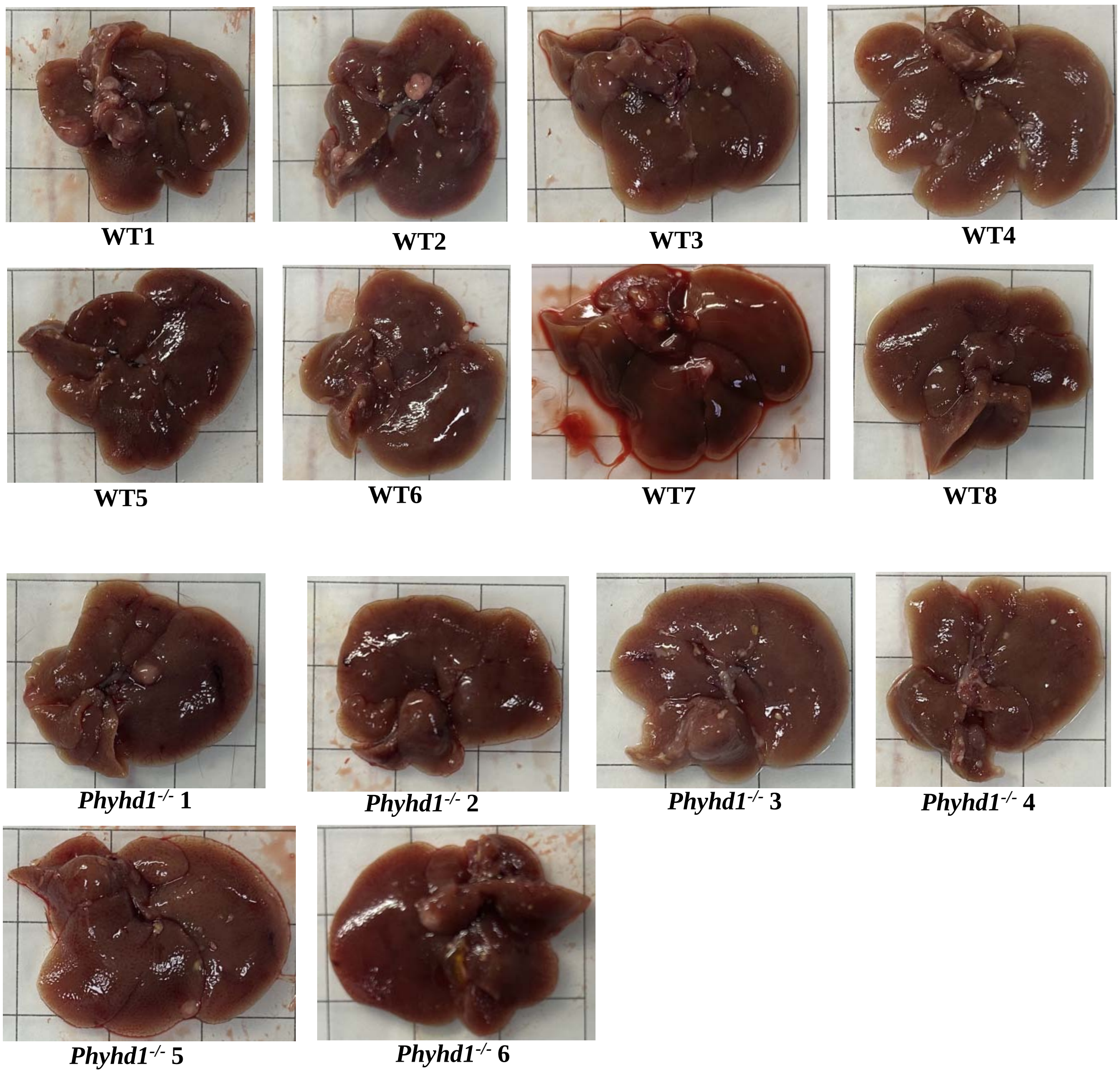

Supplement: Supplementary file 3 [file DataSheet1.PDF]
